# RETRACTED ARTICLE: Systematic Assessment of Research on Autism Spectrum Disorder and Mercury Reveals Conflicts of Interest and the Need for Transparency in Autism Research

**DOI:** 10.1007/s11948-015-9713-6

**Published:** 2015-10-27

**Authors:** Janet K. Kern, David A. Geier, Richard C. Deth, Lisa K. Sykes, Brian S. Hooker, James M. Love, Geir Bjørklund, Carmen G. Chaigneau, Boyd E. Haley, Mark R. Geier

**Affiliations:** 1 Institute of Chronic Illnesses, Inc., 14 Redgate Court, Silver Spring, MD 20905 USA; 2 Nova Southeastern University, Fort Lauderdale, FL USA; 3 CoMeD, Inc., Silver Spring, MD USA; 4 Simpson University, Redding, CA USA; 5 Council for Nutritional and Environmental Medicine, Mo i Rana, Norway; 6 University of Kentucky, Lexington, KY USA

Based on an assessment by the Editors, the Conflict of Interest statement of this article is inadequate because it fails to disclose conflicts of interest in addition to the declaration that “the authors have been involved in vaccine/biologic litigation.” In particular, Janet Kern is a board member of CONEM (Council for Nutritional and Environmental Medicine) and Geir Bjorklund is that organization’s founder and President. Mark Geier and David Geier do work under the auspices of the non-profit Institute for Chronic Illnesses, Inc. Lisa Sykes, Mark Geier and David Geier are officers of the Coalition for Mercury-free Drugs (CoMeD, Inc). Richard Deth is on the scientific advisory board of the National Autism Association. Brian Hooker is on the board of Focus for Health. James Love has been involved in amalgam litigation. Boyd Haley is involved in the development of a mercury-chelating agent. Some of the authors have a personal as well as a professional interest in autism. In addition, some authors are or have been involved in litigation related to vaccines and autism. Furthermore, the article itself contains a number of errors, and mistakes of various types that raise concerns about the validity of the conclusion. As a result, this article is being retracted by the editors without the agreement of the authors. The online version of this article contains the full text of the retracted article as electronic supplementary material.

## Electronic supplementary material

Below is the link to the electronic supplementary material.

Supplementary material 1 (PDF 557 kb)

