# Supplementary material for: RETRACTED ARTICLE: Systematic Assessment of Research on Autism Spectrum Disorder and Mercury Reveals Conflicts of Interest and the Need for Transparency in Autism Research
Source: Sci Eng Ethics. 2015 Oct 27;23(6):1689–90. doi: 10.1007/s11948-015-9713-6 (PMC5705728; doi:10.1007/s11948-015-9713-6)
Supplement: Supplementary file 1 — Supplementary material 1 (PDF 557 kb) [file 11948_2015_9713_MOESM1_ESM.pdf]

Sci Eng Ethics
DOI 10.1007/s11948-015-9713-6

REVIEW PAPER

Systematic Assessment of Research on Autism Spectrum Disorder and Mercury Reveals Conflicts of Interest and the Need for Transparency in Autism Research

Janet K. Kern[1] · David A. Geier[1] · Richard C. Deth[2] · Lisa K. Sykes[3] · Brian S. Hooker[4] · James M. Love[3] · Geir Bjørklund[5] · Carmen G. Chaigneau[3] · Boyd E. Haley[6] · Mark R. Geier[1]

Received: 31 August 2015 / Accepted: 19 October 2015
© The Author(s) 2015. This article is published with open access at Springe[illegible]ink[illegible]m

RETRACTED ARTICLE

Abstract Historically, entities with a vested interest in a [illegible]oduct that critics have suggested is harmful have consistently used researc[illegible] back their claims that the product is safe. Prominent examples are: tobacco, le[illegible], [illegible]isphenol A, and atrazine. Research literature indicates that about 80–90 % of st[illegible]dies *with* industry affiliation found no harm from the product, while only [illegible]bout 10–20 % of studies *without* industry affiliation found no harm. In pa[illegible]llel [illegible]o other historical debates, recent studies examining a possible relat[illegible]onship [illegible]etween mercury (Hg) exposure and autism spectrum disorder (ASD) [illegible] a si[illegible]ilar dichotomy. Studies sponsored and

✉ Janet K. Kern
jkern@dfwair.net

David A. Geier
davidallengeier@co[illegible]net

Richard C. De[illegible]
rdeth@nov[illegible]ed[illegible]

Lisa K. [illegible]yk[illegible]
syk[illegible]5@veri[illegible]n.net

[illegible]rian S. [illegible]oker
b[illegible]ook[illegible]r@simpsonu.edu

[illegible]s M. Love
jlove@titushillis.com

Geir Bjørklund
bjorklund@conem.org

Carmen G. Chaigneau
mamadelchinito@gmail.com

Boyd E. Haley
behaley@ctiscience.com

Mark R. Geier
mgeier@comcast.net

supported by industry or entities with an apparent conflict of interest have most often shown no evidence of harm or no "consistent" evidence of harm, while studies *without* such affiliations report positive evidence of a Hg/autism association. The potentially causal relationship between Hg exposure and ASD differs from other toxic products since there is a broad coalition of entities for whom a conflict of interest arises. These include influential governmental public health entities, the pharmaceutical industry, and even the coal burning industry. This review includes a systematic literature search of original studies on the potential relationship between Hg and ASD from 1999 to date, finding that of the studies *with* public health and/or industry affiliation, 86 % reported no relationship between Hg and ASD. However, among studies *without* public health and/or industry affiliation, only 19 % had no relationship between Hg and ASD. The discrepancy in these results suggests a bias indicative of a conflict of interest.

Keywords Research · Conflict of interest · Transparency · Autism · Mercury · Toxicants

Introduction

A possible link between exposure to mercury (Hg) and autism spectrum disorder (ASD) is a recent example of a heated debate over the association between a pervasive toxic exposure and a prevalent and devastating diagnosis. The heated debate began in the late 1990s, when it was suggested that exposure to Hg in vaccines was a risk factor for ASD ([illegible]sey 1999). In this case, as in those before it, whenever there has been a possible link between illness and a toxic exposure, there has been heated debate characterized by adamant denial.

Denial of a toxicant in disease causation can be attributed in part and firstly, to a natural inclination to resist unpleasant theories. A historical illustration of this was acrodynia (also known as Pink Disease). This almost forgotten disease, mostly affecting infants and young children, is a well-studied example of human Hg poisoning (Bjørklund 1995). Hg as the cause of acrodynia was first suggested in 1846, and again in 1922 (Hanson and Pleva 1991). Warkany and Hubbard (1948) from the United States (US) demonstrated Hg involvement in 25 out of 28 cases of acrodynia in 1948. When Hg-containing teething powders were withdrawn from the market in Australia in 1953 (followed later by the US), there was a dramatic fall in the incidence and mortality rate from acrodynia (Bjørklund 1995). However, the

RETRACTED ARTICLE

[1] Institute of Chronic Illnesses, Inc., 14 Redgate Court, Silver Spring, MD 20905, USA

[2] Nova Southeastern University, Fort Lauderdale, FL, USA

[3] CoMeD, Inc., Silver Spring, MD, USA

[4] Simpson University, Redding, CA, USA

[5] Council for Nutritional and Environmental Medicine, Mo i Rana, Norway

[6] University of Kentucky, Lexington, KY, USA

role of Hg as the primary source of acrodynia was not universally accepted even as late as 1956 (Dathan and Harvey 1965). Throughout these decades of heated debate, the science indicating acrodynia was caused by Hg exposure was resisted because, as stated by a historian studying the social and medical aspects of the illness, "poisoning was not a fashionable diagnosis" (Dally 1997).

Resistance to linking a toxic exposure to an illness can also result from a concern for liability. The responsibility for an illness or disability that results from a toxic product is borne by the industry that manufactures it, and resistance to a link between illness and an antecedent exposure is often driven by an industry that fears the consequences, since it is in their best interest to do so (Brownell and Warner 2009; McComas 2008; Friedman and Richter 2005; Sass 2006; Bridbord and Hanson 2009; Ong and Glantz 2001; Hayes 2004). As a result, conflicts of interest enter these debates and the resulting discourse is often marred by misleading information, which, unfortunately, often includes misleading assertions concerning the state of scientific research.

Historically, entities with a vested interest in a product that critics have suggested is harmful have consistently used research to back their claims that a product is safe. Brownell and Warner (2009) reviewed the issue of conflicts of interest in research. They concluded that industry manipulates research information to buy loyalty, instill doubt about criticisms, confuse the public, give ammunition to political allies, and stall or influence government action. This practice, as noted by Brownell and Warner (2009) and many other experts on the subject, continues to the present day (Barnoya and Glantz 2006; Mars and Ling 2008; Schick and Glantz 2007; Michaels 2008; Kessler 2001; Mooney 2006; Brownell and Warner 2009). The following sections provide examples of research conflicts of interest, starting with tobacco, because much of what we have learned regarding the influencing or "buying" of scientists is from tobacco litigation.

Past and Current Examples of Research Conflict of Interest and Outside Influences

Tobacco

The tobacco industry spent significant funds in their attempts to undermine the science with respect to a link between smoking and lung disease (including issues related to secondhand smoke), calling into doubt the research that showed harm from smoking (McGarity and Wagner 2008; Tong and Glantz 2007). Evidence indicates that the industry paid prominent scientists to conduct studies with the intent of countering potentially damaging scientific evidence (Cummings et al. 2007). Internal documents revealed that the industry devised a plan to become a major sponsor of medical research (Draft Recommendations for Cigarette Manufacturers 1953). According to Brandt (2012) this "call for new research" was intended to: (1) give the impression that existing studies were inadequate or flawed, referring to them as "junk" science, and (2) to create uncertainty about the harm from tobacco while making the industry appear to be a committed participant in the

RETRACTED ARTICLE

scientific endeavor (Brownell and Warner 2009). The industry developed research programs that offered funds directly to university-based scientists that would enlist their support and develop their financial dependence (Brandt 2012).

Lead

Another example is lead (Pb) poisoning. Pb poisoned the environment for decades while being used in many products (gasoline, paint, and pipes) before action was taken. The first documented case of childhood Pb poisoning in the US was in 191[illegible] By 1930, Pb paint was regulated or banned in most European countries. Nevertheless, the US lead industry fought federal and local regulation [illegible] its products and promoted its use for several more decades (Grist et al. 2004).

The toxicity of Pb was the subject of heated debate, and the debate was again marked by resistance, conflicts of interest, and misleading information. As reported by Bridbord and Hanson (2009), the Pb industry used their public relations capabilities to advertise the benefits of their products to the general public while casting doubt on the possibility of harm associated with its use. This was achieved, "…in large part, by being the primary supporter of research on health effects of lead and relying upon the scientists that it supported to communicate and interpret this research to the government and the public." (Bridbord and Hanson 2009; Sykes et al. 2014). The misinformation from research on this issue may have influenced policy-makers, because policymakers pursued [illegible] strategy that focused on diagnosing children after they were poisoned (the effect) rather than identifying toxic sources (the cause), which in effect allowed children [illegible] be Pb exposed and Pb poisoned for years to come (Grist et al. 2004).

Methylmercury

Minamata disease provid[illegible] another example of a heated debated about the link between toxic expo[illegible] and the resulting diagnosis. Minamata disease was caused by methyl-Hg poisoning, where the putative source of the Hg was methyl-Hg-cysteine from contaminated fish. The fish were contaminated with methyl-Hg from the dumping [illegible] Hg-tainted waste into water in Minamata, Japan by the Chisso Plant. The di[illegible]se wa[illegible] attributed to many other causes: infection, explosions, etc., with some of [illegible] alternative theories being promoted by the Chisso Plant itself, the company ultimately found to be responsible for the exposure (Takeuchi et al. 1978). Ea[illegible] studies conducted by the Chisso Plant found their industrial waste caused the disease; however, that information was not published. In fact, even while knowing this information, the Chisso Plant funded research into alternative causes of the disease, other than its own waste (Encyclopedia of the Earth 2009).

Atrazine

Reports of conflicts of interest in research include the herbicide atrazine, which was banned in the European Union in 2004, but is still used in the US (European Commission 2004). Hayes (2004) found that financial sponsorship was a strong

RETRACTED ARTICLE

predictor of study outcome for atrazine research ($p = 0.009$). Thus funding sources varied for studies reporting adverse effects (including government and industry funding), but all of the studies that failed to detect adverse effects were funded by the manufacturer of atrazine.

Bisphenol A

Similar to atrazine research, vom Saal and Hughes (2005), who studied bisphenol A (BPA), found that no BPA industry-funded studies have ever reported significant effects from low doses of BPA, although >90 % of government-funded studies reported significant effects from low doses of BPA. Moreover, some of the industry-funded BPA studies that reported no significant effects used a strain of rat that was inappropriate for the study of estrogenic responses (vom Saal and Hughes 2005).

Olestra

Similar results have been reported in food research. For example, among studies supportive of the fat substitute olestra, 80 % were funded by the food industry; however, in contrast, only 21 % of neutral studies and 11 % of studies critical of olestra have been funded by the industry. All authors affiliated with the maker of olestra have published studies that were supportive of olestra (Levine et al. 2003).

Conflicts of Interest in Mercury Exposure and Autism Research

Conflicts of interest in studies examining Hg exposure and the resulting risk of ASD have been noted (DeSoto and Hitlan 2010). In parallel to other historical debates over potential toxicants and their resulting adverse effects, studies examining the Hg-autism link that were sponsored and supported by entities with apparent conflicts of interest, often show no evidence of harm or no "consistent" evidence of harm from Hg exposure, even in the most vulnerable subjects, human fetuses and infants.

For example, studies on Hg exposure from coal-burning plants conducted by researchers *without* industry affiliation consistently show that Hg exposure from coal burning is a significant risk factor for ASD (Palmer et al. 2006, 2009; Blanchard et al. 2011; Windham et al. 2006). In contrast, Lewandowski et al. (20[illegible]), researchers *with* industry affiliation, examined the relationship between Hg release in Texas and ASD and found different results. Dr. Lewandowski works for Gradient, a product defense consulting firm that has received substantial sums from companies to write reports defending products such as cigarettes and BPA (Keim 2007). Lewandowski et al. (2009) concluded that Hg emissions are not "consistently" associated with ASD prevalence in Texas school districts.

Another example of such conflict of interest in research is found in studies conducted on the safety of RhoD immune globulin (RhoGam). Different formulations of Thimerosal (49.55 % Hg by weight)-containing RhoGam were routinely administered to Rh-negative mothers in the US prior to 2002. Studies conducted by

RETRACTED ARTICLE

researchers *without* industry affiliations found significant increases in maternal Rh-negativity among children with neurodevelopmental disorders (NDs), including ASD (Geier and Geier 2007a, b; Geier et al. 2008; Holmes et al. 2003). However, Johnson & Johnson, a manufacturer of RhoGam, approached Dr. Judith Miles and her department at the University of Missouri with a significant grant to help defend them from litigation. The industry-sponsored study by Miles and Takahashi (2007) commenced and concluded that exposure to ethylmercury (or Thimerosal) from RhoGam was not associated with ASD (Miles and Takahashi 2007).

In ASD, the stakes for industry are particularly high, with millions of children affected globally (DeSoto and Hitlan 2010). As mentioned, more than one industry views this issue through the lens of their own potential culpability, the coal burning industry which expels mercury into the air and the pharmaceutical industry which uses Hg as a preservative in some vaccines. However, the issue of conflicts of interest in research that examines the relationship between Hg exposure and ASD is different from the typical toxicants and products previously mentioned in that the public health sector, a powerful and influential global and governmental alliance, views this issue through the lens of its own potential culpability. According to internal documents, public health officials are concerned that negative information about Thimerosal (the Hg-based preservative used in some vaccines), if substantiated, might damage the vaccine program, for which the public health system has a vested interest in vaccine distribution and uptake (Association of American Physicians and Surgeons, Inc. 2005). As reported by the United States Congressional Report of 2003 in regard to the issue of Thimerosal and ASD, "Our public health agencies' failure to act is indicative of institutional malfeasance for self-protection and misplaced protectionism of the pharmaceutical industry." (Burton 2003).

Additionally, the public health entity, the US Centers for Disease Control and Prevention (CDC), receives millions of dollars in industry gifts and funding, including substantial support from the pharmaceutical industry (Lenzer 2015; Smith et al. 2012). According to Lenzer (2015), numerous manufacturers give donations to the CDC through the CDC Foundation. For example, in 2012–2013 Janssen donated $1.5 million, and in 2011–2012 contributors included Merck ($915,149), Genzyme ($762,000), Sanofi Aventis ($600,000), and Abbott Laboratories ($550,000) (Lenzer 201[illegible]; Smith et al. 2012). This significant financial relationship further amplifies the potential for conflict of interest on the part of the CDC.

The issue of conflict of interest in ASD can be illustrated by an examination of the published scientific literature. A systematic literature search of original studies from 1999 to date (August 2015) using the search terms "autism and mercury" reveals evidence of this bias. The results of this search are listed and briefly described in Tables 1 and 2. Table 1 shows the studies on Thimerosal (or mercury) and ASD which were sponsored or co-sponsored by those *with* public health, pharmaceutical industry, or coal burning affiliation, which represent a conflict of interest. Table 2 shows the studies on Thimerosal (or mercury) and ASD that were conducted by independent researchers *without* public health or industry affiliation.

Similar to historical debates about other toxicants, the findings reveal, that research with an apparent conflict of interest shows a bias toward the null hypothesis

RETRACTED ARTICLE

RETRACTED ARTICLE

Table 1 Studies that examined the relationship between Thimerosal (or Hg) and autism that were sponsored or co-sponsored by public health and/or had industry affiliation; 12/14 = 86 % failed to reject the null hypothesis, i.e., no relationship between Hg and ASD

Study	Evaluated	Conclusion	Affiliation with public health or industry	Found effect
Verstraeten et al. (2003)<br>Pediatrics	Assessed the possible toxicity of TCVs [illegible] infants	No analyses found significant increased risks for autism	Yes<br>Public health	No
Madsen et al. (2003)<br>Pediatrics	TCVs in Denmark and incidence of autism	Data do not support a correlation between TCVs and autism	Yes<br>Public health and industry[a]	No
Stehr-Green et al. (2003)<br>Am J Pre Med	TCVs and autism	No correlation between TCVs and autism	Yes<br>Public health and industry[a]	No
Hviid et al. (2003)<br>JAMA	To determine whether vaccination with a TM-containing vaccine is associated with autism	Results do not support a causal relationship between TCVs and ASD	Yes<br>Public health and industry[a]	No
Andrews et al. (2004)<br>Pediatrics	Relationship between the amount of TM an infant receives via DTP or DT vaccine and NDs (autism)	No evidence of an association with TM exposure	Yes<br>Public health	No
Price et al. (2010)<br>Pediatrics	TCVs and autism	No findings of increased risk for any of the ASD outcomes	Yes<br>Public health	No
Yau et al. (2014)<br>Environ Res	Prenatal and early-life exposures to Hg	Total Hg in serum collected from mothers during mid-pregnancy and newborn bloodspots were not significantly associated with ASD	Yes<br>Public health	No
Windham et al. (2006)<br>Environ Health Perspect	ASD and environmental exposures, ambient air, San Francisco Bay	Increased risk of ASD associations included Hg, cadmium, nickel, trichloroethylene, and vinyl chloride	Yes<br>Public health	Yes
Schechter and Grether (2008)<br>Arch Gen Psychiatry	Autism prevalence in California after removal of TM from most childhood vaccines	Data do not support the hypothesis that exposure to TCVs during childhood is a primary cause of autism	Yes<br>Public health	No

RETRACTED ARTICLE

Table 1 continued

Study	Evaluated	Conclusion	Affiliation with public health or industry	Found effect
De Palma et al. (2012)<br>J Aut Dev Disord	H[illegible] toxic me[illegible]ls in autism versus controls	Found no association between autism and hair Hg	Yes<br>Public health	No
Wright et al. (2012)<br>PLoS One	Urinary [illegible]g l[illegible]els between children with ASD [illegible]nd contro[illegible] normal ($n = 121$) a[illegible] with [illegible]earning disabilities ($n =$ 3[illegible]	No statistically significant differences were found between children with ASD and controls	Yes<br>Public health	No
Dickerson et al. (2015)<br>Sci Total Environ	ASD prevalence and prox[illegible]ty to industrial facilities relea[illegible]ing arsenic, lead or Hg	Association between urban residential proximity to industrial facilities emitting air pollutants and higher ASD prevalence	Yes<br>Public health	Yes
Miles and Takahashi (2007)<br>Am J Med Genet A	Association between Rh status, RhoGam use in pregnancy and autism	[illegible] association was found between ma[illegible]ernal RhoGam use and autism	Yes<br>Industry[b]	No
Lewandowski et al. (2009)<br>J Toxicol Environ Health A	Hg exposure from coal-fired power plants and autism in Texas	An[illegible]sis su[illegible]gests Hg emissions not [illegible]onsiste[illegible]y associated with autism pre[illegible]ce in Texas school districts	Yes<br>Industry[c,d]	No

TCVs Thimerosal-containing vaccines, *RhoGAM* Rho(D) immune globulin, *ASD* autism spectrum [illegible]sorder, *TM* Thimerosal, *Hg* mercury

[a] Statens Serum Institut (Danish vaccine manufacturer)

[b] Johnson and Johnson Pharmaceutical Company (RhoGam manufacturer)

[c] Gradient, a product defense consulting firm that has received substantial sums from companies to wri[illegible]e[illegible] defending products such as cigarettes and BPA (Keim 2007)

[d] Electric Power Research Institute

RETRACTED ARTICLE

Table 2 Studies that ex[illegible]ed the [illegible]lationship between Thimerosal (or Hg) that were conducted by independent researchers *without* public health or industry affiliation; 12/62 = 19 % failed to rej[illegible]t the [illegible] hypothesis, i.e., no relationship between Hg and ASD

Study/Journal	Evaluated	Conclusion	Affiliation with public health or industry	Found effect
Rose et al. (2015)<br>J Toxicol	Hum[illegible] autism versus controls expose[illegible]o TM	Autism LCLs exhibited greater reduction in ATP-linked respiration, maximal respiratory capacity, and reserve capacity, compared to control LCLs	No	Yes
Geier et al. (2015)<br>Biol Trace Elem Res	Risk of a PDD fo[illegible]owi[illegible] TM exposure from Hib	Cases of autism/PDD were significantly more likely to have had TM exposure from Hib	No	Yes
Geier et al. (2014b)<br>J Biochem Pharmacol Res	Risk of a ND following TM e[illegible]sure from DTaP	Cases of autism were significantly more likely to have had TM exposure from DTaP	No	Yes
Geier et al. (2014a)<br>Int J Environ Res Public Health	Dose dependent relationship betwe[illegible] TM exposure and NDs	Cases of autism/PDD more likely than controls, per microgram of TM exposure	No	Yes
Alabdali et al. (2014)<br>Behav Brain Func	Concentration of two toxic heavy metals, lead and Hg were measured in red blood cells, plus glutathione-s-transferase (GST) and vitamin E	[illegible]SD had significantly higher Pb and Hg levels and lower GST activity and vitamin E conc[illegible]rations compared with the c[illegible]ntro[illegible] The levels of heavy metals (Hg and l[illegible]), GS[illegible]nd vitamin E were correla[illegible]ed [illegible] th[illegible] severity of the social and cogn[illegible]ve [illegible]pairment measures	No	Yes
Macedoni-Lukšič et al. (2015)<br>Biol Trace Elem Res	levels of metals in blood aluminum, lead, Hg in ASD compared to children with neurological disorders	No significant [illegible]fference[illegible] blood levels of metals between the[illegible]oups was found	No	No
Geier et al. (2013)<br>Transl Neurodegener	Thimerosal-containing vaccine administration as a risk factor for ASD in VAERS and VSD	Cases of autism were sig[illegible]ica[illegible]more likely to have had TM expo[illegible]re fro[illegible] HepB	No	Yes

Table 2 continued

Study/Journal	Evaluated	Conclusion	Affiliation with public health or industry	Found effect
Sharpe et al. (2013)<br>J Toxicol	Human lymphocytes in autism versus controls	Autism families showed TM hypersensitivity, none of control individuals displayed this response, the TM concentration required to inhibit cell proliferation in these individuals was only 40 % of controls	No	Yes
Albizzati et al. (2012)<br>Przegl Epidemiol	Metals in blood, urine and hair samples from children with autism and children with neuropsychiatric disorders, unspecified	No difference was found between children with autism and children with neuropsychiatric disorders, unspecified	No	No
Abdullah et al. (2012)<br>J Aut Dev Disord	Heavy metals in children's tooth enamel	No significant differences in levels of these neurotoxicants for children with ASDs compared with TD children	No	No
Geier et al. (2012)<br>Int J Environ Res Public Health	Hair toxic metal concentrations and ASD severity	Increasing hair Hg concentrations significantly correlated with increased ASD severity	No	Yes
Rahbar et al. (2013)<br>Neurotox Res	Investigate the association between blood Hg concentrations in children and ASDs	Found no association between blood Hg concentrations in children and ASDs	No	No
Adams et al. (2013)<br>Biol Trace Elem Res	Investigated both the level of toxic metals in children with autism and the possible association of those toxic metals with autism severity in whole blood, RBCs, and urine	Found a strong association of levels of toxic metals with variation in the degree of severity of autism for all the severity scales, with cadmium (whole blood) and Hg (whole blood and RBC) were the most consistently significant variables	No	Yes
van Wijngaarden et al. (2013)<br>Epidemiology	evaluated the association between prenatal methylHg exposure and ASD phenotype	Prenatal exposure to methylHg was not associated with ASD phenotypic behaviors	No	No

RETRACTED ARTICLE

RETRACTED ARTICLE

Table 2 continued

Study/Journal	Evaluated	Conclusion	Affiliation with public health or industry	Found effect
Yasuda and Tsutsui (2013)<br>Sci Rep	Hair co[illegible]ntra[illegible]tions of 26 trace elements in chi[illegible]en [illegible] autistic disorders	Individuals had high burden of aluminum, cadmium and lead, and 2.8 % or less from Hg and arsenic burden	No	Yes
Blaucok-Busch et al. (2012)<br>Maedica (Buchar)	Examined if DMS[illegible] treatment reduced heavy metal bu[illegible]n and symptoms in ASD	Levels of cadmium, Hg, and lead were reduced and ASD symptoms showed improvements	No	Yes
Blaurock-Busch et al. (2012)<br>Maedica (Buchar)	Assessed the levels o[illegible] ten toxic [illegible]etals and essential elements in [illegible]air samp[illegible] of children with autism, and to [illegible]rela[illegible]e the level of these elements with [illegible]e seve[illegible] of autism	Elevated hair concentrations were noted for aluminum, arsenic, cadmium, Hg, antimony, nickel, lead, and vanadium in autism versus controls	No	Yes
Hodgson et al. (2014)<br>Exp Biol Med (Maywood)	Investigated redox and methylation metabolites, level of protein homocysteinylation and hair Hg levels [illegible] autism and controls	Hg levels were markedly elevated in the hair of autistic subjects versus control subjects; glutathione in autistic subjects was significantly below control levels, while levels of homocysteine and S-a[illegible]en[illegible]ylhomocysteine were elevated	No	Yes
Stamova et al. (2011)<br>Neurotox Res	Correlations between gene expression and Hg levels in blood of boys with and without autism	Finding[illegible] [illegible]uggest [illegible]different genetic transc[illegible]ption[illegible] pro[illegible]rams associated with Hg in au[illegible] [illegible]ared to controls	No	Yes
Blaurock-Busch et al. (2011)<br>Maedica (Buchar)	Exposure to Hg and other heavy metals in children with autism spectrum disorder versus controls	Statistically sig[illegible]ficant d[illegible]erences in the mean urine levels o[illegible] [illegible]umi[illegible]um, barium, cerium, Hg, and le[illegible]	No	Yes
Obrenovich et al. (2011)<br>Biol Trace Elem Res	Hair toxic metals in autism versus controls	Abnormal markers of thi[illegible]etal[illegible]m, as well as a significant alterati[illegible] in deposition of several heavy [illegible]etal speci[illegible]s, particularly arsenic, Hg, coppe[illegible], and [illegible]n in hair samples between the grou[illegible]	No	Yes

RETRACTED ARTICLE

Table 2 continued

Study/Journal	Evaluated	Conclusion	Affiliation with public health or industry	Found effect
Shandley and Austin (2011)<br>J Toxicol Environ Health A	To test the hypothesis that individuals with a known Hg sensitivity to Hg (pink disease survivors) may be more likely to have descendants with an ASD	Prevalence rate of ASD among the grandchildren of pink disease survivors (1 in 22) to be significantly higher than the comparable general population prevalence rate (1 in 160)	No	Yes
Lakshmi Priya and Geetha (2011)<br>Biol Trace Elem Res	Lead and Hg in hair and nails autism versus controls	Significant elevation in the levels of toxic metals lead and Hg in both hair and nail samples in autism versus controls	No	Yes
Geier et al. (2010)<br>Acta Neurobiol Exp (Warsaw)	Blood Hg levels in autism and controls	Hg levels were 1.9-fold significantly increased among subjects diagnosed with an ASD (21.4 microg/L) in comparison to controls (11.4 microg/L)	No	Yes
Hertz-Picciotto et al. (2010)<br>Environ Health Perspect	Blood Hg levels in autism versus controls	After accounting for dietary and other differences in Hg exposures, total Hg in blood not statistically different	No	No
Majewska et al. (2010)<br>Acta Neurobiol Exp	Levels of hair Hg in autism versus controls	Autistic children significantly differed from healthy peers in the concentrations of Hg in hair	No	Yes
Gallagher and Goodman (2010)<br>J Toxicol Environ Health A	Association between TM-containing HepB vaccination of male neonates and autism	Threefold greater odds for autism diagnosis	No	Yes
James et al. (2009)<br>FASEB J	LCLs derived from autistic children and controls, effects of TM on and GSH levels	TM resulted in greater [illegible]crease in GSH/GSSG ratio and increase in free radical generation in autism versus control cells	No	Yes
Palmer et al. (2009)<br>Health Place	Power plant emissions and autism	Every 1000 pounds of industrial release, there was a corresponding 2[illegible] % increase in autism rates and a 3.7 % increase associated with power plant emissions	No	Yes

RETRACTED ARTICLE

Table 2 continued

Study/Journal	Evaluated	Conclusion	Affiliation with public health or industry	Found effect
Geier et al. (2009)<br>Acta Neurobiol Exp	Materna[illegible]ent[illegible]l amalgams and autism sev[illegible]ty	Subjects with > or = 6 amalgams were 3.2-fold significantly more likely to be diagnosed with autism (severe) in comparison to ASD (mild) than subjects with < or = 5 amalgams	No	Yes
Young et al. (2008)<br>J Neuro Sci	Ecological study [illegible]f T[illegible]ontaining vaccines and risk of NDs	Increased risk of an ASD diagnosis with TCVs	No	Yes
Geier et al. (2008)<br>Neuro Endocrin Lett	Maternal Rh-negativity/[illegible]M-cont[illegible]g RhoGAM	Increase in ASD with maternal Rh-negativity	No	Yes
Geier and Geier (2007a)<br>J Matern Fetal Neonatal Med	Maternal Rh-negativity/TM-contai[illegible] RhoGAM	Increase in ASD with maternal Rh-negativity	No	Yes
Geier and Geier (2007b)<br>J Toxicol Environ Health A	Regressive autism and TM exposure	S[illegible]gnificant dose–response relationship [illegible]etween the severity of the regressive ASDs and total Hg dose children received from TC[illegible]s/RhoGAM	No	Yes
Zhang and Wong (2007)<br>Environ Int	Examined Hg exposure increases in China	Ev[illegible]ence[illegible]uggests an increase in autism relate[illegible]o incr[illegible]ing Hg exposure	No	Yes
Adams et al. (2007)<br>J Toxicol Environ Health A	Level of Hg, lead, and zinc in baby teeth in autism versus controls	Children w[illegible]had significantly (2.1-fold) higher l[illegible]els o[illegible]	No	Yes
Soden et al. (2007)<br>Clin Toxicol (Phila)	24-Hour provoked urine excretion test for heavy metals in children with autism	Excess chelatable bod[illegible]urd[illegible] of As, Cd, Pb, or Hg is zero	No	No
DeSoto and Hitlan (2007)<br>J Child Neurol	Re-analysis of Ip study data (mentioned below)	Significant relation does ex[illegible]betw[illegible] the blood levels of Hg and ASD; in the [illegible]astic gr[illegible]up,<br>Severity of autism was invers[illegible] related[illegible] hair Hg levels	No	Yes

RETRACTED ARTICLE

Table 2 continued

Study/Journal	Evaluated	Conclusion	Affiliation with public health or industry	Found effect
Walker et al. (2006)<br>Neurotoxicology	Heat sh[illegible] pr[illegible]tein transcripts and MT exp[illegible]ed [illegible]M in autism versus controls	No apparent differences between autistic and non-autistic sibling responses in this very small sampling group	No	No
Singh and Hanson (2006)<br>Pediatr Allergy Immunol	Metallothion[illegible] (M[illegible]) and anti-MT in autism and controls ex[illegible]sed to TCVs	MT and anti-MT were no different suggesting no TM induced MT-autoimmunity in autism	No	No
Palmer et al. (2006)<br>Health Place	Hg release, special ed[illegible]cation [illegible], and autism disorder	Association between environmentally released Hg and special education rates were fully mediated by increased autism rates	No	Yes
Al-Ayadhi (2005)<br>Neurosciences (Riyadh)	Hair metals in autism versus cont[illegible]	Higher levels of toxic heavy metals Hg, lead, arsenic, antimony and cadmium in autistic [illegible]pectrum disorders as compared to the [illegible]ontrols	No	Yes
Geier and Geier (2006)<br>J Toxicol Environ Health A	Dose (50 vs. 25 micrograms) of Hg from TM in VAERS	Increased odds ratios for autism with higher doses of [illegible]M	No	Yes
Fido and Al-Saad (2005)<br>Autism	Toxic metals in the hair of children with autism differ from age- and sex-matched healthy controls	Childre[illegible] [illegible]ith autism had significantly ($p <$ [illegible]001) [illegible]r in-hair concentration levels of [illegible], H[illegible] [illegible]nd uranium.	No	Yes
Geier and Geier (2005)<br>Med Sci Monit	Association between TCVs DTaP comparison to TM-free DTaP and autism in VAERS and VSD	Exposure to H[illegible] [illegible]rom TC[illegible]s administered in the US was a [illegible]onsist[illegible] [illegible]ificant risk factor for autism	No	Yes
Geier and Geier (2003a)<br>Pediatr Rehabil	Dose of TCVs and autism in VAERS and USDE data	Dose–response curves sho[illegible] increases in odds ratios of NDs (au[illegible]m) [illegible]n both VAERS and USDE closely [illegible]nearly correlated with increasing do[illegible]es of T[illegible] containing childhood vaccines	No	Yes

RETRACTED ARTICLE

Table 2 continued

Study/Journal	Evaluated	Conclusion	Affiliation with public health or industry	Found effect
Geier and Geier (2003b)<br>Exp Biol Med	TM-DT[illegible] an[illegible] NDs in VAERS	An association was found between TM-DTaP and autism	No	Yes
Vojdani et al. (2003)<br>Int J Immunopathol Pharmacol	Measure[illegible] IgG, Ig[illegible] and IgA antibodies against C[illegible]26, [illegible]69, streptokinase, gliadin and cas[illegible] pept[illegible]s and against ethyl Hg bound to h[illegible]an serum albumin in autism	TM binds to lymphocyte receptors and/or tissue enzymes, resulting in autoimmune reaction in children with autism	No	Yes
Ip et al. (2004)<br>J Child Neurol	Hair and blood Hg leve[illegible] and a[illegible]	No difference in the mean Hg levels	No	No
Singh and Rivas (2004)<br>J Biomed Sci	A study of Hg-induced antinuclear [illegible] antilaminin antibodies in autistic [illegible]nd normal children who had been pre-administered with TCVs	Serum level of these two autoimmune markers did not significantly differ between autistic and normal children	No	No
Geier and Geier (2004)<br>Med Sci Monitor	Hg doses from TCVs on population prevalence of autism	[illegible]vidence showing a direct relationship between increasing doses of Hg from TC[illegible] [illegible] autism	No	Yes
Blanchard et al. (2011)<br>Rev Environ Health	Occurrence of autism as related to distribution of Hg in ambient air	Risk of [illegible]tism is greater in the geographic areas [illegible] high[illegible] le[illegible]els of ambient Hg	No	Yes
Mrozek-Budzyn et al. (2011)<br>Przegl Epidemiol.	To determine an association of TCVs exposure with the risk of autism	No evidenc[illegible] of [illegible] ass[illegible]iation between TCVs and au[illegible]sm	No	No
Holmes et al. (2003)<br>Int J Toxicol	Relationship between autism and hair Hg levels	Hg levels statisticall[illegible] [illegible]ifferent [illegible]om controls and correlated with symp[illegible] severity. Mothers in the autistic [illegible]oup [illegible]d significantly higher levels o[illegible] Hg exp[illegible]sure through RhoGam and amalg[illegible]m filling[illegible]	No	Yes

Table 2 continued

Study/Journal	Evaluated	Conclusion	Affiliation with public health or industry	Found effect
Mostafa and AL-Ayadhi (2015)<br>Clin Cell Immunol	Blood [illegible] lev[illegible]ls and seropositivity of anti-MB[illegible] au[illegible]bodies in autistic children	Serum levels of blood Hg were significantly higher in autistic children than healthy controls; increased levels of blood Hg were found in 48 % of autistic patients, and 72 % of autistic children had anti-MBP auto-antibodies. There was a significant positive association between the elevated levels of blood Hg and anti-MBP auto-antibodies in autistic children	No	Yes
Yassa (2014)<br>Environ Toxicol Pharmacol	Blood and hair samples fro[illegible] child[illegible] from Upper Egypt with autism, 2–[illegible] years of age and 45 controls in the sa[illegible] age range	High level of Hg and lead among those kids with autism, with significant decline in the blood level of lead and Hg with the use of DMSA as a chelating agent	No	Yes
Khan et al. (2014)<br>J Physiol Pharmacol	Brain Hg levels measured in extracortica[illegible] regions autism versus controls	[illegible]ain Hg levels measured in extracortical regions in children with autism versus controls were not different	No	No
Roberts et al. (2013)<br>Environ Health Perspect	Associations between US EPA-modeled levels of hazardous air pollutants at time and place of birth and ASD	Overa[illegible] [illegible]asure of metals were significantly assoc[illegible]d with ASD, with odds ratios rang[illegible] from [illegible] (for overall metals measure) [illegible] ([illegible] diesel and Hg)	No	Yes
Mostafa and Refai (2007)<br>Egypt J Pediatr Allergy Immunol	Serum antineuronal<br>antibodies and blood Hg levels were estimated between autism and controls	Higher seropos[illegible]vity for [illegible]ntineuronal antibodies and highe[illegible] [illegible] Hg in autism versus controls. S[illegible]positivi[illegible] of antineuronal antibodies h[illegible] positive association with elevat[illegible] [illegible] Hg (found in 70 % of autistic children) [illegible]oth ma[illegible]kers positively associated with be[illegible]avioral abnormalities, autistic regression, E[illegible] abnormalities	No	Yes

RETRACTED ARTICLE

Table 2 continued

Study/Journal	Evaluated	Conclusion	Affiliation with public health or industry	Found effect
Biamonte et al. (2014)<br>Neurotoxicology	Mice exposed to MeHg during the prenatal and early postnatal period, either at subtoxic dose or at toxic dose	Higher MeHg dose caused dramatic reduction of PCs in all mice and "autism-like" features (loss of sociability, preference for sameness) in genetically susceptible mice	No	Yes
Bradstreet et al. (2003)<br>J Am Phys Surgeons	Children with ASD and controls treated with multiple doses of DMSA	Children with ASD excreted six-fold greater Hg than controls	No	Yes
DeSoto and Hitlan (2012)<br>J Environ Protection	Examined Hg-related fish advisories and rate of autism	Hg-related fish advisories are found to be a strong predictor of a state's autism rate, r = 0.48, $p < 0.001$	No	Yes

ASD autism spectrum disorder, *ND* neurodevelopmental disorder, *LCL* lymphoblastoid cell lines, *TM* Thimerosal, *PDD* pervasive developmental disorder, *Hib* haemophilus influenzae type b vaccine, *DTaP* diphtheria, tetanus, acellular pertussis, *He*[illegible] hepatitis B vaccine, *RhoGAM* Rho(D) immune globulin, *VAERS* vaccine adverse events reporting system, *VSD* vaccine safety datalink, *USDE* US Department of Education, *GSH* glutathione, *TCVs* Thimerosal-containing vaccines, *MT* metallothionein, *DMSA* dimercaptosuccinic acid, *anti-MBP* anti-myelin basic protein, *EPA* Environmental Protection Agency, *RBC* red blood cells, *MeHg* methylHg, *PCs* purkinje cells, *TD* typically developing, *DMSA* 2,3-dimercaptosuccinic acid, *Hg* mercury

or "no effect" (i.e., no relationship between Hg and ASD). Of the studies *with* public health or industry affiliation, 86 % (12/14) failed to reject the null hypothesis (Table 1). However, of the studies *without* public health or industry affiliation, only 19 % (12/62) failed to reject the null hypothesis. In other words, over 80 % of the studies *without* public health or industry affiliation found evidence a relationship between Hg exposure and ASD (Table 2). The dramatic discrepancy in these results, 86 versus 19 %, provides evidence of biased outcomes, indicative of a conflict of interest.

The Need for Transparency in Autism Research

As mentioned earlier, the stakes in the ASD debate are high. In the past two decades, there has been a dramatic increase in ASD rates, with a [illegible] % increase from 4.2 per 1000 in 1996 to 15.5 per 1000 in 2010 (Van Naarden Braun et al. 2015). ASD is considered to have reached epidemic proportions and is an issue of high national and international concern. The critical importance of this debate only heightens the urgent need for transparency in autism research.

Transparency in autism research, including access to research datasets used, would provide for the review and evaluation of studies and the partiality or impartiality which characterized them, and encourage a system of checks and balances. When study findings are deemed inaccurate and/or biased, transparency in autism research would allow for either confirmation or correction. For instance, in 2004, Ip et al. published a study comparing blood and hair Hg levels in children with ASD and controls, reporting that there was no difference in the mean Hg levels. However, other scientists noted that there did appear to be a significant difference in the mean Hg levels between the groups and subsequently requested the data. Upon re-analysis, the data revealed that there was indeed a significant difference in the mean mercury levels between children with ASD and controls (DeSoto and Hitlan 2007). The authors of the re-analysis stated: "If there is any link between autism and mercury, it is absolutely crucial that the first reports of the question are not falsely stating that no link occurs."

As another instance, Dr. Polly R. Sager, Division of Microbiology and Infectious Disease, National Institute of Allergy and Infectious Disease (NIADI), US National Institutes of Health (NIH), made a presentation, "NIAID Studies on Thimerosal" to the Institute of Medicine of the US National Academy of Sciences on February 9, 200[illegible] (IOM 2004). In her presentation, she presented crucial evidence on the comparative distribution and persistence of Hg in the brain and blood following methyl-Hg and Thimerosal administration to infant monkeys mimicking the US childhood vaccine schedule of the 1990s by other investigators (Burbacher et al. 2005). It was later discovered following Dr. Sager's presentation that she misrepresented data on the true extent that Hg distributed and persisted in the monkey brain following Thimerosal administration. She was eventually forced to supply in her own words, "Corrected Slide Submitted to IOM May 3, 2004". However, even with the corrections, errors still were present in the "Corrected Slide" and the information still did not reflect that data ultimately published by the

RETRACTED ARTICLE

study investigators (Burbacher et al. 2005). As a consequence, the IOM, in evaluating the relationship between Thimerosal exposure and ASD risk, was unable to consider accurate and true data as to the distribution and persistence of Hg in the monkey brain following Thimerosal administration mimicking the US childhood vaccine schedule of the 1990s.

Examples of Studies that Illustrate the Importance of Transparency in Autism Research

A number of ASD and Hg studies, sponsored by entities with an apparent conflict of interest, appear to have arrived at questionable conclusions. Moreover, the authors of these studies have, unfortunately, failed to make their datasets available to others for further evaluation, exemplifying the need for transparency in autism research.

As expressed by Baskin and Gross (2015), editors of the journal *Neurology* on the need for greater transparency in research in general: "The responsibility for promoting greater openness in research falls not only to the individuals performing the work, but to the funders of the work (including government, foundation, and industry sponsors), institutions where the work is being done, and to journal editors and peer reviewers, who do the final check on the quality of the research before it is released to readers." The following examples illustrate this point.

Verstraeten et al. (2000, 2003)

In the late 1990s, in a study sponsored by the CDC, Verstraeten et al. (2000) "categorized the cumulative ethyl-Hg exposure from [T]himerosal[-]containing vaccines after one month of life and assessed the subsequent risk of degenerative and developmental neurologic disorders and renal disorders before the age of six." The authors applied proportional hazard models adjusting for HMO, year of birth, and gender, and they excluded premature babies. The original reported results showed that the relative risk (RR) of developing a neurologic development disorder was 1.8 [95 % confidence intervals (CI) 1.1–2.8] when comparing the highest exposure group at 1 month of age (cumulative dose >25 μg) to the unexposed group. Similarly, they "…also found an elevated risk for the following disorders: autism (RR 7.6, 95 % CI 1.8–31.5), non-organic sleep disorders (RR 5.0, 95 % CI 1.6–15.9), and speech disorders (RR 2.1, 95 % CI 1.1–4.0)" in the highest exposure group. The results were presented to the Epidemic Intelligence Service Annual Conference, CDC in Atlanta, GA, in 2000, but it remained as an abstract and was never published as a full paper (Verstraeten et al. 2000).

However, the subsequently published results from this study diverged from the aforementioned results presented in 2000 (Bernard 2004; Put Children First 2006). In the published study, Verstraeten et al. (2003) concluded that "No consistent significant associations were found between TCVs and neurodevelopmental outcomes" When the dataset was requested by independent researchers with the help of the United States Congress, the dataset, according to the CDC was "destroyed" (personal communication, Stuart Burns on behalf of Congressman

Weldon 2003). Explaining the unavailability of this dataset, the Institute of Medicine (IOM) stated: “Analytic data files from some previously published VSD studies had not been archived in a standard manner, so it was difficult to respond expeditiously to requests to reanalyze published VSD studies.” (IOM 2005).

Yau et al. (2014)

In 2013 Yau et al., in conjunction with the California Department of Public Health, submitted a study to the *Journal of Autism and Developmental Disorders*, entitled “Prenatal and neonatal peripheral blood Hg levels and autism spectrum disorders.” The study was rejected by reviewers because the results did not match the conclusions. The conclusion stated by the authors was that the “Results indicate that levels of total mercury in serum collected from mothers during mid-pregnancy and from newborn bloodspots were not significantly associated with risk of ASD.” However, in Table 2 of the study, the geometric mean maternal serum Hg concentrations between the general population (0.32) and the ASD group (0.48) had a P value of 0.05. Thus, maternal serum blood Hg levels were significantly higher in the ASD group than in the general population, although the study authors failed to state this. The study was published later in the journal *Environmental Research* without addressing this issue. To date, the California Department of Public Health (CDPH), University of California at Davis (UC Davis), Kaiser Permanente, as well as individual authors, have failed to release the study dataset for further evaluation despite receiving numerous requests for this information. The CDPH stated that they did not have the complete dataset and that they are required to make sure the data are destroyed after the studies are over (personal communication, Dr. Martin Kharrazi, CPDH, 6/25/2015). UC Davis refused to release the dataset claiming, “researcher’s privilege, based upon a strong Constitutional interest in the right of scholars to conduct research without interference, an aspect to the academic freedom recognized as a special concern of the First Amendment.’” (Personal communication, Michele M. McCuen, Legal Analyst, Office of the Campus Counsel, Office of the Chancellor and Provost, University of California, 8/21/2015). Kaiser Permanente refused to release the dataset claiming, “The Freedom of Information Act (FOIA) only applies to federal agencies. It does not apply to an institution like [illegible].” (Personal communication, Caroline Milner, National Research Compliance Officer, National Compliance in Research Program, Kaiser Foundation Research Institute, 8/7/2015).

Uno et al. (2015)

In 2015, Uno et al. published a study in the journal *Vaccine* investigating the relationship between the risk of ASD and early exposure to the combined Measles–Mumps–Rubella (MMR) vaccine and the relationship between Thimerosal from vaccinations in Japanese children. The authors concluded that there were no significant differences in the timing of MMR vaccination or Thimerosal dosage between cases and controls for any age group. However, there was a statistical error that nullified the conclusions offered by the authors. This error was found in Table 2

RETRACTED ARTICLE

at 24 months of age. From the values provided in Table 2 of the study (see below), it was evident that the difference between cases and controls at 24 months was indeed statistically significant with a high degree of confidence. Thus, there was a statistically significant yet unacknowledged relationship between Thimerosal exposure and the risk of ASD. The journal *Vaccine* was notified of the error.

From the originally provided data, the following results were documented for children age 24 months: Unpaired *t* test mean of * sample 1 from summary data = 804.2 (n = 189) mean of * sample 2 from summary data = 632.1 (n = 224).

Assuming equal variances, the combined standard error = 71.8, *df* = 41[illegible] t = 2.40 one sided *p* = 0.0085 two sided *p* = 0.017, 95 % confidence interval for difference between means = 30.88–313.32 power (for 5 % significan[illegible]) = 90.07 %.

Assuming unequal variances, the combined standard error = 72.06, *d*[illegible] = 39[illegible].17, t(d) = 2.39 one sided *p* = 0.0087 two sided *p* = 0.0174, 95 % co[illegible]nce [illegible]terval for difference between means = 30.45–313.75 power (for 5[illegible] sign[illegible]ficance) = 66.35 %.

When the journal *Vaccine* was made aware of the stati[illegible]tic[illegible]l error, it notified the authors. In response to this notification, the authors chang[illegible] [illegible] data values (mean and standard deviation) for the controls in Table 2 a[illegible] 24 mon[illegible]s from 632.1 (715.1) to 676.8 (719.5) in their study, as summarized in Fig. 1 [illegible]us, the data was changed in order to maintain the original and erroneous [illegible]onclusion. In essence, the conclusion determined the data, rather th[illegible] [illegible] data determining the conclusion. However, no explanation for the error or [illegible]stific[illegible]tion for the change was given. To date, the journal *Vaccine* and the stu[illegible]y auth[illegible] have refused requests to release the study dataset for further evaluati[illegible]. [illegible]thou[illegible]h there was never a response from any of the study authors, the journa[illegible] [illegible]accine stated (in response to the notification of the

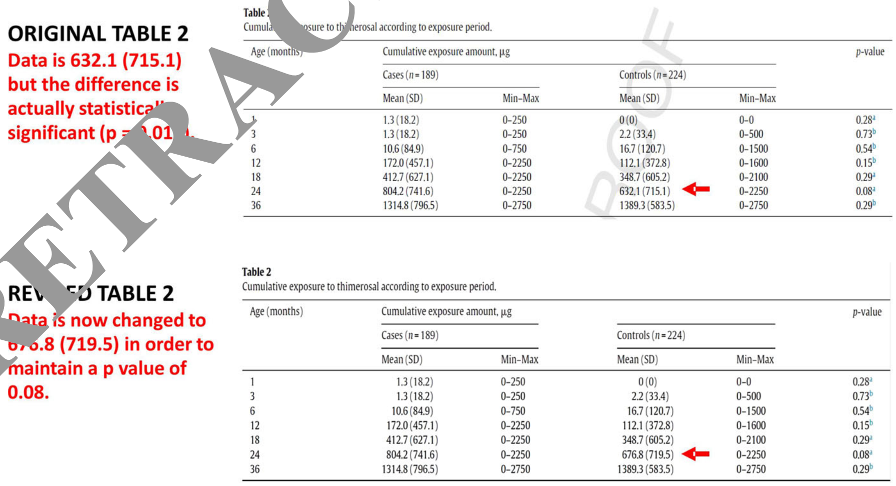

Table [illegible]
Cumula[illegible]osure to th[illegible]erosal according to exposure period.

Age (months)	Cumulative exposure amount, µg				p-value
	Cases (n = 189)		Controls (n = 224)		
	Mean (SD)	Min–Max	Mean (SD)	Min–Max	
1	1.3 (18.2)	0–250	0 (0)	0–0	0.28[a]
3	1.3 (18.2)	0–250	2.2 (33.4)	0–500	0.73[b]
6	10.6 (84.9)	0–750	16.7 (120.7)	0–1500	0.54[b]
12	172.0 (457.1)	0–2250	112.1 (372.8)	0–1600	0.15[b]
18	412.7 (627.1)	0–2250	348.7 (605.2)	0–2100	0.29[a]
24	804.2 (741.6)	0–2250	632.1 (715.1)	0–2250	0.08[a]
36	1314.8 (796.5)	0–2750	1389.3 (583.5)	0–2750	0.29[b]

Table 2
Cumulative exposure to thimerosal according to exposure period.

Age (months)	Cumulative exposure amount, µg				p-value
	Cases (n = 189)		Controls (n = 224)		
	Mean (SD)	Min–Max	Mean (SD)	Min–Max	
1	1.3 (18.2)	0–250	0 (0)	0–0	0.28[a]
3	1.3 (18.2)	0–250	2.2 (33.4)	0–500	0.73[b]
6	10.6 (84.9)	0–750	16.7 (120.7)	0–1500	0.54[b]
12	172.0 (457.1)	0–2250	112.1 (372.8)	0–1600	0.15[b]
18	412.7 (627.1)	0–2250	348.7 (605.2)	0–2100	0.29[a]
24	804.2 (741.6)	0–2250	676.8 (719.5)	0–2250	0.08[a]
36	1314.8 (796.5)	0–2750	1389.3 (583.5)	0–2750	0.29[b]

Fig. 1 Changing data in Table 2 from Uno et al. (2015) after the authors were notified of a statistical error. Changing the data allowed the authors to maintain their conclusion of no association between ASD and Thimerosal

error and the request for the dataset) that, "Following the feedback from your group, the authors have made a minor correction to Table 2 and an acknowledgement thereof is made in the article. We are grateful for sharing your observations with us. As appropriate action has been undertaken, we now consider this matter to be resolved." (Personal communication, Alina Helsloot, Executive Publisher Immunology and Microbiology, Elsevier and Dr. Gregory Poland, Editor in Chief, Vaccine, 4/9/2015).

Curtis et al. (2015)

In 2015 Curtis et al., including employees from public health, published a study titled: "Examination of the Safety of Pediatric Vaccine Schedules in a Non-Human Primate Model: Assessments of Neurodevelopment, Learning, and Social Behavior." The study examined the safety of pediatric vaccine schedules in a non-human primate model, and they concluded that the results, "…provided no consistent evidence of neurodevelopmental deficits or aberrant behavior in vaccinated animals." However, the data presented in the study did not match the conclusions. As one researcher (Dr. Dan Laks) stated on PubMed Commons: "Supplementary Figure 5 clearly shows a drastic reduction in learning in the Thimerosal exposed group. The authors' discussion: 'In the present study animals in the TCV group appeared to perform poorer than controls in learning set testing but showed little evidence that their responses had organized into a strategy that was different from that of the control group. In fact, the reported difference was only found in the overall mean averaged across all of the blocks and trials, not in their learning across trials or blocks, which is the outcome needed to indicate a strategy difference.' But in fact, a deficit in learning seems to be in multiple groups, for if one looks at group E, there seems to be a slope difference from the control signifying a key difference between exposures for learning strategy. These results are not reported. Perhaps Supplemental Figure 5 results should have been the title of this study instead: 'Ethylmercury from vaccines reduces learning capacity.'"

Based on the clear difference between the data and the conclusions, a request for the dataset was submitted. To date, the University of Texas Southwestern Medical Center (UTSW), the Johnson Center for Child Health and Development, as well as individual authors of the study, have failed to release the study dataset for further evaluation. UTSW stated that because Dr. Hewitson, the lead researcher on the study, was volunteer faculty, they did not "…have any influence or control over her or her data." (Personal communication, Carol Tamminga, MD, UTSW, 5/8/2015). The Johnson Center for Child Health & Development stated that, "Given the size and scope of the project (and the multiple data sets involved) it would be a great undertaking to appropriately and adequately coordinate another review of these data sets. It would require the ability to read the data (requiring the acquisition of several specific, proprietary software licenses), the adequate and appropriate personnel to interpret the data (requiring PhD level statisticians and PhD level scientists with experience in interpreting discrimination learning and social behavior in animals), and it would require a significant amount of funding to cover not only these items, but also the significant amount of time that would be required of the statisticians and

RETRACTED ARTICLE

researchers who completed this original project in order to review and have knowledge of the procedures of the study and the scope of the data sets (as well as the time involved to extract the data).” (Personal communication, Anissa Ryland, Executive Director, The Johnson Center for Child Health & Development, 4/2/2015).

Summary and Conclusion

Historically, entities/industries with a vested interest in a product whose safety is in dispute have consistently used research to back their claims that a product is safe. The effects of a funding source on research outcomes have been examined, and it has been shown that industry or responsible entity affiliated studies are far more likely to yield outcomes favorable to that industry/entity (Boone et al. 2014). When this conflict of interest influences research, the resulting scientific debate on products, toxicants, etc. can be confounded by misleading information. Indeed, this is precisely the outcome desired by the sponsors of such conflicted research.

A conflict of interest in autism research has been noted, particularly when examining Hg exposure and the risk of ASD (DeSoto and Hitlan 2010). However, conflicts of interest in this debate are different from other cases because not only industries (e.g., the coal-burning industry and the pharmaceutical industry), but also public health institutions view this issue through the lens of their own potential culpability. Further complicating the matter is the fact that public health entities often control access to the relevant datasets. Indeed, a systematic examination of the research literature in the Hg-autism debate shows that research funded by these conflicted entities is more likely to yield conclusions favorable to that industry/entity, finding no relationship between Hg exposure and the risk of ASD.

Transparency in autism research is of utmost importance. The current examples of studies offering questionable conclusions clearly illustrate the need for openness and accountability. ASD is an issue of high national and international concern, where the stakes are high and researchers and policymakers need to be cognizant of the issue of conflicts of interest in autism research (DeSoto and Hitlan 2010).

One way of achieving improved openness and transparency in autism research would be for authors, journals, and funding sources to require greater openness and data sharing. As mentioned, the responsibility for promoting greater openness in research falls not just to the authors, but to the funders, institutions, and journal editors (Baskin and Gross 2015). The examples provided in this analysis suggest that some authors, journals, and institutions could improve in the area of helping to promoting greater openness.

The Proceedings of the National Academy of Sciences of the United States of America (PNAS) has developed and adopted standards concerning the responsibilities of authorship in the biological sciences. It is referred to as the Uniform Principle for Sharing Integral Data and Materials Expeditiously or “UPSIDE”. In October of 2001, National Academies committee evaluated the responsibilities of authors to share data and materials referenced in their publications, the role of journals to impose requirements for data and material sharing, and whether a

RETRACTED ARTICLE

common set of requirements for sharing does or should exist (Cozzarelli 2001, 2004; Cech 2003). They established that authors are obligated to release data and materials to enable others to verify or replicate published findings. They stated that one "upside" to this is it keeps science honest (Cozzarelli 2001, 2004; Cech 2003). In an article by Nicholas R. Cozzarelli, Editor-in-Chief of PNAS, he described some of the comments of the board members and these comments may be relevant to the current discussion (Cozzarelli 2001). Two of the comments are as follows:

> I am one of the few people here who represents the private sector at this point, and I would love to be able to publish in prestigious journals and withhold the data. But I think it is wrong.
> Scientific journals should play no role in the protection of the private in[illegible]es[illegible] of authors, or in shielding data from the community. Protection is far [illegible]field of the mission of journals, and shielding is antithetical to it.

Identifying causative factors for ASD is already a challe[illegible]ing t[illegible]k for the scientific community, demanding the highest standards of ope[illegible]ess and transparency. Any departure from these standards represents [illegible] di[illegible]service to all.

Acknowledgments This study was supported by the non-profit [illegible]01(c)(3) I[illegible]itute of Chronic Illnesses, Inc., and the non-profit 501(c)(3) CoMeD, Inc.

Compliance with Ethical Standards

Conflict of interest There are no competing fina[illegible]l in[illegible]rests. The authors have been involved in vaccine/biologic litigation.

Open Access This article is distrib[illegible]ed unde[illegible]he terms of the Creative Commons Attribution 4.0 International License (http://creative[illegible]on[illegible]ns.org/licenses/by/4.0/), which permits unrestricted use, distribution, and reproduction in [illegible] medi[illegible], provided you give appropriate credit to the original author(s) and the source, provi[illegible]e a link to the Creative Commons license, and indicate if changes were made.

RETRACTED ARTICLE

References

Abdullah[illegible]M. M., [illegible]A. R., Goldberg, W. A., Clarke-Stewart, K. A., Dudgeon, J. V., Mull, C. G., et al. (2[illegible]12[illegible]Heavy metal in children's tooth enamel: Related to autism and disruptive behaviors? *Journal of Autis[illegible]nd Developmental Disorders, 42*(6), 929–936.

A[illegible]am[illegible] J. B., Audhya, T., McDonough-Means, S., Rubin, R. A., Quig, D., Geis, E., et al. (2013). Toxi[illegible]ological status of children with autism vs. neurotypical children and the association with a[illegible]ism severity. *Biological Trace Element Research, 151*(2), 171–180.

[illegible]ms, J. B., Romdalvik, J., Ramanujam, V. M., & Legator, M. S. (2007). Mercury, lead, and zinc in baby teeth of children with autism versus controls. *Journal of Toxicology and Environmental Health Part A, 70*(12), 1046–1051.

Alabdali, A., Al-Ayadhi, L., & El-Ansary, A. (2014). A key role for an impaired detoxification mechanism in the etiology and severity of autism spectrum disorders. *Behavioral and Brain Functions, 10*, 14.

Al-Ayadhi, L. Y. (2005). Heavy metals and trace elements in hair samples of autistic children in central Saudi Arabia. *Neurosciences (Riyadh)., 10*(3), 213–218.

Albizzati, A., Morè, L., Di Candia, D., Saccani, M., & Lenti, C. (2012). Normal concentrations of heavy metals in autistic spectrum disorders. *Minerva Pediatrica, 64*(1), 27–31.

Andrews, N., Miller, E., Grant, A., Stowe, J., Osborne, V., & Taylor, B. (2004). Thimerosal exposure in infants and developmental disorders: A retrospective cohort study in the United Kingdom does not support a causal association. *Pediatrics, 114*, 584–591.

Association of American Physicians and Surgeons, Inc. (2005). *Selected vaccine authorities from CDC, FDA, and manufacturers discuss, in a closed meeting, the possibility of neurodevelopment disorders resulting from vaccine components*. http://www.aapsonline.org/vaccines/cdcfdaexperts.htm. Accessed 05/26/2015.

Barnoya, J., & Glantz, S. A. (2006). The tobacco industry's worldwide ETS Consultants project: European and Asian components. *The European Journal of Public Health, 16*(1), 69–77.

Baskin, P. K., & Gross, R. A. (2015). *Transparency in research and reporting: Expanding the effort through new tools for authors and editors*. Wolters-Kluwer Author Newsletter; Author Resour[illegible] Review. Accessed 8/17/2015.

Bernard, S. (2004). Association between thimerosal-containing vaccine and autism. *Journal [illegible]of the American Medical Association, 291*(2), 180. **(author reply 180–181)**.

Biamonte, F., Latini, L., Giorgi, F. S., Zingariello, M., Marino, R., De Luca, R., [illegible]t al. (20[illegible]. Associations among exposure to methylmercury, reduced Reelin expression, and [illegible]ender i[illegible] the cerebellum of developing mice. *Neurotoxicology, 45*, 67–80.

Bjørklund, G. (1995). Mercury and acrodynia. *The Journal of Orthomolecular M[illegible]10*, 145–146.

Blanchard, K. S., Palmer, R. F., & Stein, Z. (2011). The value of ecologic studi[illegible]ercury [illegible]oncentration in ambient air and the risk of autism. *Reviews on Environmental Health, 26*([illegible]111–118.

Blaucok-Busch, E., Amin, O. R., Dessoki, H. H., & Rabah, T. (2012). [illegible]cy of [illegible]MSA therapy in a sample of Arab children with autistic spectrum disorder. *Maedic[illegible]7*(3), 214–221.

Blaurock-Busch, E., Amin, O. R., Dessoki, H. H., & Rabah, T. (2012[illegible]Toxic metals and essential elements in hair and severity of symptoms among children [illegible]ith autis[illegible]. *Maedica (Buchar)., 7*(1), 38–48.

Blaurock-Busch, E., Amin, O. R., & Rabah, T. (2011). Heavy metal[illegible] and trace elements in hair and urine of a sample of Arab children with autistic spectru[illegible]disorder. *[illegible]aedica (Buchar)., 6*(4), 247–257.

Boone, M. D., Bishop, C. A., Boswell, L. A., Brod[illegible]nan, [illegible]D., Burger, J., Davidson, C., et al. (2014). Pesticide regulation amid the influence of indu[illegible]. *Bi[illegible]Science, 64*(10), 917–922.

Bradstreet, J., Geier, D. A., Kartzinel, J. J., Adams, J. [illegible]Geier, M. R. (2003). A case-control study of mercury burden in children with autis[illegible]sp[illegible]tru[illegible]m disorders. *Journal of the American Physicians and Surgeons, 8*, 76–79.

Brandt, A. M. (2012). Inventing co[illegible] of int[illegible]est: A history of tobacco industry tactics. *American Journal of Public Health, 10*[illegible](1), 63[illegible]

Bridbord, K., & Hanson, D. A. [illegible]2009). Pers[illegible]nal perspective on the initial federal health-based regulation to remove lead from gaso[illegible]e. *Env[illegible]ronmental Health Perspectives, 117*(8), 1195–1201.

Brownell, K., & Warner, K. E. [illegible]). The perils of ignoring history: Big tobacco played dirty and millions died. How [illegible] is big food? *The Milbank Quarterly, 87*(1), 259–294.

Burbacher, T. M., Shen, D. [illegible], Liberato, N., Grant, K. S., Cernichiari, E., & Clarkson, T. (2005). Compariso[illegible] blood and brain mercury levels in infant monkeys exposed to methylmercury or vaccines [illegible] thimerosal. *Environmental Health Perspectives, 113*(8), 1015–1021.

Burton, D[illegible] O. [illegible]03). US congressional report. Mercury in medicine report. In Committee on G[illegible]ment R[illegible]form (Ed.). *Congressional record* (pp. E1011–E1030). United States Congress.

Cech[illegible]T. R. [illegible]03) *Sharing publication-related data and materials: Responsibilities of authorship in the [illegible]ife scien[illegible]es*. www.nap.edu/books/0309088593/html. Accessed 08/28/2015.

C[illegible]relli[illegible]R. (2001). Unfettered access to published results. *Proceedings of the National Academy of [illegible]nces of the United States of America, 98*, 8159.

[illegible]ozzarelli, N. R. (2004). UPSIDE: Uniform principle for sharing integral data and materials expeditiously. *Proceedings of the National Academy of Sciences of the United States of America, 101*(11), 3721–3722.

Cummings, K. M., Brown, A., & O'Connor, R. (2007). The cigarette controversy. *Cancer Epidemiology, Biomarkers and Prevention, 16*, 1070–1076.

Curtis, B., Liberato, N., Rulien, M., Morrisroe, K., Kenney, C., Yutuc, V., et al. (2015). Examination of the safety of pediatric vaccine schedules in a non-human primate model: Assessments of neurodevelopment, learning, and social behavior. *Environmental Health Perspectives, 123*(6), 579–589.

Dally, A. (1997). The rise and fall of pink disease. *Social History of Medicine, 10*(2), 291–304.

RETRACTED ARTICLE

Dathan, J. G., & Harvey, D. D. (1965). Pink disease—10 years after (the epilogue). *British Medical Journal, 1*(5443), 1181–1182.

De Palma, G., Catalani, S., Franco, A., Brighenti, M., & Apostoli, P. (2012). Lack of correlation between metallic elements analyzed in hair by ICP-MS and autism. *Journal of Autism and Developmental Disorders, 42*(3), 342–353.

Desoto, M. C., & Hitlan, R. T. (2007). Blood levels of mercury are related to diagnosis of autism: A reanalysis of an important data set. *Journal of Child Neurology, 22*(11), 1308–1311.

Desoto, M. C., & Hitlan, R. T. (2010). Sorting out the spinning of autism: Heavy metals and the question of incidence. *Acta Neurobiologiae Experimentalis, 70*(2), 165–176.

DeSoto, M. C., & Hitlan, R. T. (2012). Fish consumption advisories and the surprising relationship to prevalence rate of developmental disability as reported by public schools. *Journal of Envir[illegible] Protection, 3*(11), 1579–1589.

Dickerson, A. S., Rahbar, M. H., Han, I., Bakian, A. V., Bilder, D. A., Harrington, R. A., et al. [illegible]2015). Atism spectrum disorder prevalence and proximity to industrial facilities releasing arsenic, [illegible] or mercury. *Science of the Total Environment, 536*, 245–251.

Draft Recommendations for Cigarette Manufacturers. (1953). *John W. Hill papers, S[illegible]te Hist[illegible]rical Society of Wisconsin, Archives Division*. http://www.ttlaonline.com/HKWIS/[illegible]96.[illegible] Accessed 11/06/2011.

Encyclopedia of the Earth. (2009). *Minamata disease*. http://www.eoearth.[illegible]/view/a[illegible]cle/154624/. Accessed 05/08/2015.

European Commission. (2004). http://eur-lex.europa.eu/legal-content/EN/[illegible]/?uri=[illegible]serv:OJ.L_.2004.078.01.0053.01.ENG. Accessed 05/12/2015.

Fido, A., & Al-Saad, S. (2005). Toxic trace elements in the hair of child[illegible] with autism. *Autism, 9*(3), 290–298.

Friedman, L., & Richter, E. D. (2005). Conflicts of interest and scien[illegible]ntegrity. *International Journal of Occupational Medicine and Environmental Health, 11*(2), [illegible]05–206.

Gallagher, C. M., & Goodman, M. S. (2010). Hepati[illegible] B vaccination of male neonates and autism diagnosis, NHIS 1997–2002. *Journal of Toxi[illegible]ology [illegible]d Environmental Health, Part A, 73*(24), 1665–1677.

Geier, D. A., Audhya, T., Kern, J. K., & Geier, M. R. [illegible]010). Differences in blood mercury levels in autism spectrum disorders; is there a [illegible]re[illegible]hold level? *Acta Neurobiologiae Experimentalis, 70*, 177–186.

Geier, D. A., & Geier, M. R. (20[illegible] An as[illegible]essment of the impact of thimerosal on childhood neurodevelopmental disorder[illegible] *Pedia[illegible] Rehabilitation, 6*(2), 97–102.

Geier, M. R., & Geier, D. A[illegible] (2003b). [illegible]eurodevelopmental disorders after thimerosal-containing vaccines: A brief comm[illegible]icatio[illegible]. *Experimental Biology and Medicine (Maywood)., 228*(6), 660–664.

Geier, D. A., & Geier, M[illegible](2004). A comparative evaluation of the effects of MMR immunization and mercury doses from [illegible]rosal-containing childhood vaccines on the population prevalence of autism. *Med[illegible] Scien[illegible]e Monitor, 10*(3), PI33–PI39.

Geier, D. A., [illegible]Ge[illegible] M. R. (2005). A two-phased population epidemiological study of the safety of thime[illegible]rosal-c[illegible]aining vaccines: A follow-up analysis. *Medical Science Monitor, 11*(4), CR160–CR1[illegible]

Geie[illegible] D. A., [illegible]Geier, M. R. (2006). An evaluation of the effects of thimerosal on neurodevelopmental [illegible]isorders reported following DTP and Hib vaccines in comparison to DTPH vaccine in the United [illegible]tates[illegible] *Journal of Toxicology and Environmental Health, Part A, 69*(15), 1481–1495.

Geie[illegible] A., & Geier, M. R. (2007a). A prospective study of thimerosal-containing Rho(D)-immune globulin administration as a risk factor for autistic disorders. *The Journal of Maternal-Fetal & Neonatal Medicine, 20*(5), 385–390.

[illegible]eier, D. A., & Geier, M. R. (2007b). A case series of children with apparent mercury toxic encephalopathies manifesting with clinical symptoms of regressive autistic disorders. *Journal of Toxicology and Environmental Health, Part A, 70*(10), 837–851.

Geier, D. A., Hooker, B. S., Kern, J. K., King, P. G., Sykes, L. K., & Geier, M. R. (2013). A two-phase study evaluating the relationship between thimerosal-containing vaccine administration and the risk for an autism spectrum disorder diagnosis in the United States. *Translational Neurodegeneration, 2*(1), 25.

Geier, D. A., Hooker, B. S., Kern, J. K., King, P. G., Sykes, L. K., & Geier, M. R. (2014a). A dose-response relationship between organic mercury exposure from thimerosal-containing vaccines and

RETRACTED ARTICLE

neurodevelopmental disorders. *International Journal of Environmental Research and Public Health, 11*(9), 9156–9170.

Geier, D. A., Kern, J. K., & Geier, M. R. (2009). A prospective study of prenatal mercury exposure from maternal dental amalgams and autism severity. *Acta Neurobiologiae Experimentalis (Wars), 69*(2), 189–197.

Geier, D. A., Kern, J. K., King, P. G., Sykes, L. K., & Geier, M. R. (2012). Hair toxic metal concentrations and autism spectrum disorder severity in young children. *International Journal of Environmental Research and Public Health, 9*(12), 4486–4497.

Geier, D. A., Kern, J. K., King, P. G., Sykes, L. K., & Geier, M. R. (2015). A case-control study evaluating the relationship between thimerosal-containing haemophilus influenzae type b vaccine administration and the risk for a pervasive developmental disorder diagnosis in the United States. *Biological Trace Element Research, 163*(1–2), 28–38.

Geier, D. A., Kern, J. K., King, P. G., Sykes, L. K., Homme, K. G., & Geier, M. R. (2014b). The risk of neurodevelopmental disorders following a thimerosal-preserved DTaP formulation in comparison to its thimerosal-reduced formulation in the vaccine adverse event reporting system (VAERS). *Journal of Biochemical Pharmacology Research, 2*(2), 64–73.

Geier, D. A., Mumper, E., Gladfelter, B., Coleman, L., & Geier, M. R. (2008). Neurodevelopmental disorders, maternal Rh-negativity, and Rho(D) immune globulins: A multi-center assessment. *Neuroendocrinology Letters, 29*(2), 272–280.

Grist staff. (2004). *Rhode Island lawsuit pinpoints lead poisoning as an environmental, not medical, problem.* http://grist.org/article/spin2/, www.grist.com. Accessed 06/1[illegible]/2015.

Halsey, N. A. (1999). *Perspective on the use of thimerosal-containing vaccines*. Presentation at the National Vaccine Advisory Committee Workshop on Thimerosal and Vaccines. Institute of Vaccine Safety website. www.vaccinesafety.edu, http://www.vaccinesafety.edu/Testimony-O99.htm. Accessed 04/25/2015.

Hanson, M., & Pleva, J. (1991). The dental amalgam issue—Review. *Experientia, 47*(1), 9–22.

Hayes, T. (2004). There is no denying this: Defusing the confusion about atrazine. *BioScience, 54*(12), 1138–1149.

Hertz-Picciotto, I., Green, P. G., Delwiche, L., Han[illegible], R., Walker, C., & Pessah, I. N. (2010). Blood mercury concentrations in CHARGE study children with and without autism. *Environmental Health Perspectives, 118*(1), 161–166.

Hodgson, N. W., Waly, M. I., Al-Farsi, Y. M., Al-Sharbati, M. M., Al-Farsi, O., Ali, A., et al. (2014). Decreased glutathione and elevated hair mercury levels are associated with nutritional deficiency-based autism in Oman. *Experimental Biology and Medicine (Maywood), 239*(6), 697–706.

Holmes, A. S., Blaxill, M. F., & Haley, B. E. (2003). Reduced levels of mercury in first baby haircuts of autistic children. *International Journal of Toxicology, 22*(4), 277–285.

Hviid, A., Stellfeld, M., Wohlfahrt, [illegible], & Melbye, M. (2003). Association between thimerosal-containing vaccine and autism. *Journal of the American Medical Association, 290*, 1763–1766.

Institute of Medicine. (2004). *Surgery PR, "NIAID studies on thimerosal"*. http://iom.nationalacademies.org/~/media/[illegible]52DCA80EA040B68D278387BC73B968.ashx. Accessed 08/21/2015.

Institute of Medicine Report. (2005). *Vaccine safety research, data access, and public trust*. http://www.iom.edu/Reports/2005/Vaccine-Safety-Research-Data-Access-and-Public-Trust.aspx. Accessed 05/2[illegible]/2015.

Ip, P., Wong, V., Ho, M., Lee, J., & Wong, W. (2004). Mercury exposure in children with autistic spectrum disorder: Case-control study. *Journal of Child Neurology, 19*(6), 431–434. **(Erratum in: J Child Neurol. 2007;22(11):1324)**.

James, S. J., Rose, S., Melnyk, S., Jernigan, S., Blossom, S., Pavliv, O., & Gaylor, D. W. (2009). Cellular and mitochondrial glutathione redox imbalance in lymphoblastoid cells derived from children with autism. *The FASEB Journal, 23*(8), 2374–2383.

Keim, B. (2007). EPA silent on air pollution experts' conflicts of interest. http://www.wired.com/2007/07/epa-silent-on-a/. Accessed 05/13/2015.

Kessler, D. A. (2001). *A question of intent: A great American battle with a deadly industry*. New York: Public Affairs.

Khan, A., Harney, J. W., Zavacki, A. M., & Sajdel-Sulkowska, E. M. (2014). Disrupted brain thyroid hormone homeostasis and altered thyroid hormone-dependent brain gene expression in autism spectrum disorders. *Journal of Physiology and Pharmacology, 65*(2), 257–272.

RETRACTED ARTICLE

RETRACTED ARTICLE

Lakshmi Priya, M. D., & Geetha, A. (2011). Level of trace elements (copper, zinc, magnesium and selenium) and toxic elements (lead and mercury) in the hair and nail of children with autism. *Biological Trace Element Research, 142*(2), 148–158.

Lenzer, J. (2015). Centers for disease control and prevention: Protecting the private good? *British Medical Journal, 350*, h2362.

Levine, J., Gussow, J. D., Hastings, D., & Eccher, A. (2003). Authors' financial relationships with the food and beverage industry and their published positions on the fat substitute olestra. *American Journal of Public Health, 93*(4), 664–669.

Lewandowski, T. A., Bartell, S. M., Yager, J. W., & Levin, L. (2009). An evaluation of surrogate chemical exposure measures and autism prevalence in Texas. *Journal of Toxicology and Environmental Health, Part A, 72*(24), 1592–1603.

Macedoni-Lukšič, M., Gosar, D., Bjørklund, G., Oražem, J., Kodrič, J., Lešnik-Musek, P., et al. (2015). Levels of metals in the blood and specific porphyrins in the urine in children with autism spectrum disorders. *Biological Trace Element Research, 163*(1–2), 2–10.

Madsen, K. M., Lauritsen, M. B., Pedersen, C. B., Thorsen, P., Plesner, A. M., Andersen, P. H., & Mortensen, P. B. (2003). Thimerosal and the occurrence of autism: Negative ecological evidence from Danish population-based data. *Pediatrics, 112*(3 Pt 1), 604–606.

Majewska, M. D., Urbanowicz, E., Rok-Bujko, P., Namyslowska, I., & Mierzejewski, [illegible] (2010). Age-dependent lower or higher levels of hair mercury in autistic children than [illegible] healthy controls. *Acta Neurobiologiae Experimentalis (Wars), 70*(2), 196–208.

Mars, S. G., & Ling, P. M. (2008). Meanings and motives: Experts debating tobacco addiction. *American Journal of Public Health, 98*(10), 1793–1802.

McComas, K. A. (2008). Session 5: Nutrition communication. The role of trust in health communication and the effect of conflicts of interest among scientists. *Proceedings of the Nutrition Society, 67*(4), 428–436.

McGarity, T., & Wagner, W. (2008). *Bending science: How special interests corrupt public health research*. Cambridge, MA: Harvard University Press.

Michaels, D. (2008). *Doubt is their product: How industry's assault on science threatens your health*. New York: Oxford University Press.

Miles, J. H., & Takahashi, T. N. (2007). Lack of association between Rh status, Rh immune globulin in pregnancy and autism. *Journal of Medical Genetics Part A, 143A*(13), 1397–1407.

Mooney, C. (2006). *Republican war on science*. New York: Basic Books.

Mostafa, G. A., & AL-Ayadhi, L. A. (2015). The possible association between elevated levels of blood mercury and the increased frequency of serum anti-myelin basic protein auto-antibodies in autistic children. *Journal of Clinical & Cellular Immunology, 6*, 2.

Mostafa, G. A., & Refai, T. M. [illegible] (2007). Antineuronal antibodies in autistic children: Relation to blood mercury. *Egyptian Journal of Pediatric Allergy and Immunology, 5*(1), 21–30.

Mrozek-Budzyn, D., Majewska, R., Kiełtyka, A., & Augustyniak, M. (2011). Lack of association between thimerosal-containing vaccines and autism. *Przeglad Epidemiologiczny, 65*(3), 491–495.

Obrenovich, M. E., Shamberger, R. J., & Lonsdale, D. (2011). Altered heavy metals and transketolase found in autistic spectrum disorder. *Biological Trace Element Research, 144*(1–3), 475–486.

Ong, E. K., & Glantz, S. A. (2001). Constructing "sound science" and "good epidemiology": Tobacco, lawyers, and public relations firms. *American Journal of Public Health, 91*(11), 1749–1757.

Palmer, R. F., Blanchard, S., Stein, Z., Mandell, D., & Miller, C. (2006). Environmental mercury release, special education rates, and autism disorder: An ecological study of Texas. *Health Place, 12*(2), 203–209.

Palmer, R. F., Blanchard, S., & Wood, R. (2009). Proximity to point sources of environmental mercury release as a predictor of autism prevalence. *Health Place, 15*(1), 18–24.

Price, C. S., Thompson, W. W., Goodson, B., Weintraub, E. S., Croen, L. A., Hinrichsen, V. L., et al. (2010). Prenatal and infant exposure to thimerosal from vaccines and immunoglobulins and risk of autism. *Pediatrics, 126*, 656–664.

Put Children First. (2006) *Chapter II: 1999–2000*. Simpsonwood. http://putchildrenfirst.org/chapter2.html. Accessed 05/30/2015.

Rahbar, M. H., Samms-Vaughan, M., Loveland, K. A., Ardjomand-Hessabi, M., Chen, Z., Bressler, J., et al. (2013). Seafood consumption and blood mercury concentrations in Jamaican children with and without autism spectrum disorders. *Neurotoxicity Research, 23*(1), 22–38.

Roberts, A. L., Lyall, K., Hart, J. E., Laden, F., Just, A. C., Bobb, J. F., et al. (2013). Perinatal air pollutant exposures and autism spectrum disorder in the children of Nurses' Health Study II participants. *Environmental Health Perspectives, 121*(8), 978–984.

Rose, S., Wynne, R., Frye, R. E., Melnyk, S., & James, S. J. (2015). Increased susceptibility to ethylmercury-induced mitochondrial dysfunction in a subset of autism lymphoblastoid cell lines. *Journal of Toxicology, 2015*, 573701.

Sass, J. (2006). Credibility of scientists: Conflict of interest and bias. *Environmental Health Perspectives, 114*(3), A147–A148.

Schechter, R., & Grether, J. K. (2008). Continuing increases in autism reported to California's developmental services system: Mercury in retrograde. *Archives of General Psychiatry, 65*(1), 19–24.

Schick, S. F., & Glantz, S. A. (2007). Old ways, new means: Tobacco industry funding of academic and private sector scientists since the master settlement agreement. *Tobacco Control, 16*, 157–[illegible]64.

Shandley, K., & Austin, D. W. (2011). Ancestry of pink disease (infantile acrodynia) identifie[illegible] as [illegible] risk factor for autism spectrum disorders. *Journal of Toxicology and Environmental H[illegible]alth, Par[illegible]*, *74*(18), 1185–1194.

Sharpe, M. A., Gist, T. L., & Baskin, D. S. (2013). B-lymphocytes from a populati[illegible] of [illegible]dre[illegible] with autism spectrum disorder and their unaffected siblings exhibit hypersen[illegible]sitivit[illegible] [illegible]o thimerosal. *Journal of Toxicolology, 2013*, 801517.

Singh, V. K., & Hanson, J. (2006). Assessment of metallothionein and antibodies [illegible] metallothionein in normal and autistic children having exposure to vaccine-derived thi[illegible]osal. *Pe[illegible]iatric Allergy and Immunology, 17*(4), 291–296.

Singh, V. K., & Rivas, W. H. (2004). Detection of antinuclear and ant[illegible]inin antibodies in autistic children who received thimerosal-containing vaccines. *J[illegible]nal of [illegible]iomedical Science, 11*(5), 607–610.

Smith, B. D., Morgan, R. L., Beckett, G. A., Falck-Ytter, Y., Hol[illegible]zman, D., Teo, C. G., et al. (2012). Recommendations for the identification of chroni[illegible] [illegible]epatitis C [illegible]virus infection among persons born during 1945–1965. *MMWR Recommendations [illegible]nd R[illegible]rts, 61*(RR-4), 1–32.

Soden, S. E., Lowry, J. A., Garrison, C. B., & [illegible]sserm[illegible]n, G. S. (2007). 24-Hour provoked urine excretion test for heavy metals in children with [illegible]ism and typically developing controls, a pilot study. *Clinical Toxicology, 45*(5), 476[illegible]

Stamova, B., Green, P. G., Tian, Y., [illegible]ertz-[illegible]ciotto, I., Pessah, I. N., Hansen, R., et al. (2011). Correlations between gene express[illegible] and m[illegible]rcury levels in blood of boys with and without autism. *Neurotoxicity Research, 19*(1[illegible]31–4[illegible]

Stehr-Green, P., Tull, P., Stellfe[illegible]d, M., Mort[illegible]nson, P. B., & Simpson, D. (2003). Autism and thimerosal-containing vaccines: Lack [illegible] consis[illegible]ent evidence for an association. *American Journal of Preventive Medicine, 25*, 101–106.

Sykes, L. K., Geier, D. [illegible]King, P. G., Kern, J. K., Haley, B. E., Chaigneau, C. G., et al. (2014). Thimerosal as discri[illegible]inati[illegible]n: Vaccine disparity in the UN Minamata convention on mercury. *Indian Journal of [illegible]cal E[illegible]hics, 11*(4), 206–218.

Takeuchi, T., [illegible] K[illegible]anag, S., & Miyajima, H. (1978). Ultrastructural changes of human sural nerves in th[illegible] neur[illegible]thy induced by intrauterine methylmercury poisoning (so-called fetal Minamata dise[illegible] *Virch[illegible]ws Archiv B Cell Pathology, 27*(2), 137–154.

Tong[illegible] E. K., [illegible] Glantz, S. A. (2007). Tobacco industry efforts undermining evidence linking secondhand [illegible]moke w[illegible]th cardiovascular disease. *Circulation, 116*(16), 1845–1854.

U[illegible] Y., U[illegible]hiyama, T., Kurosawa, M., Aleksic, B., & Ozaki, N. (2015). Early exposure to the combined [illegible]sles–mumps–rubella vaccine and thimerosal-containing vaccines and risk of autism spectrum disorder. *Vaccine, 33*(21), 2511–2516.

Va[illegible] Naarden Braun, K., Christensen, D., Doernberg, N., Schieve, L., Rice, C., Wiggins, L., et al. (2015). Trends in the prevalence of autism spectrum disorder, cerebral palsy, hearing loss, intellectual disability, and vision impairment, metropolitan Atlanta, 1991–2010. *PLoS ONE, 10*(4), e0124120.

van Wijngaarden, E., Davidson, P. W., Smith, T. H., Evans, K., Yost, K., Love, T., et al. (2013). Autism spectrum disorder phenotypes and prenatal exposure to methylmercury. *Epidemiology, 24*(5), 651–659.

Verstraeten, T., Davis, R. L., DeStefano, F., Lieu, T. A., Rhodes, P. H., Black, S. B., et al. (2003). Safety of thimerosal-containing vaccines: A two-phased study of computerized health maintenance organization databases. *Pediatrics, 112*(5), 1039–1048.

RETRACTED ARTICLE

Verstraeten, T., Davis, R. L., Gu, D., & DeStefano, F. (2000). Increased risk of developmental neurologic impairment after high exposure to thimerosal-containing vaccine in first month of life. In *Epidemic intelligence service annual conference* (Vol. 49). Atlanta, GA: Centers for Disease Control and Prevention.

Vojdani, A., Pangborn, J. B., Vojdani, E., & Cooper, E. L. (2003). Infections, toxic chemicals and dietary peptides binding to lymphocyte receptors and tissue enzymes are major instigators of autoimmunity in autism. *International Journal of Immunopathology and Pharmacology, 16*(3), 189–199.

vom Saal, F. S., & Hughes, C. (2005). An extensive new literature concerning low-dose effects of bisphenol A shows the need for a new risk assessment. *Environmental Health Perspectives, 113*(8), 926–933.

Walker, S. J., Segal, J., & Aschner, M. (2006). Cultured lymphocytes from autistic children and no[illegible] autistic siblings up-regulate heat shock protein RNA in response to thimerosal challenge. *Neurotoxicology, 27*(5), 685–692.

Warkany, J., & Hubbard, D. M. (1948). Mercury in the urine of children with acrodynia. *Lance[illegible] 1*[illegible]09), 829.

Windham, G. C., Zhang, L., Gunier, R., Croen, L. A., & Grether, J. K. (2006). Autism spec[illegible]um dis[illegible]rders in relation to distribution of hazardous air pollutants in the San Francisco bay [illegible] *E[illegible]ron[illegible]nental Health Perspectives, 114*(9), 1438–1444.

Wright, B., Pearce, H., Allgar, V., Miles, J., Whitton, C., Leon, I., et al. (2012) [illegible]ompari[illegible]on of urinary mercury between children with autism spectrum disorders and control childr[illegible] *PLoS ONE, 7*(2), e29547.

Yassa, H. A. (2014). Autism: A form of lead and mercury toxicity[illegible] *En[illegible]ental Toxicology and Pharmacology, 38*(3), 1016–1024.

Yasuda, H., & Tsutsui, T. (2013). Assessment of infantile m[illegible]ral imba[illegible]ances in autism spectrum disorders (ASDs). *International Journal of Environmental Re[illegible]n and Public Health, 10*(11), 6027–6043.

Yau, V. M., Green, P. G., Alaimo, C. P., Yoshida, C. [illegible] Lutsky, M., Windham, G. C., et al. (2014). Prenatal and neonatal peripheral blood mercu[illegible]y lev[illegible] and autism spectrum disorders. *Environmental Research, 133*, 294–303.

Young, H. A., Geier, D. A., & Geier, M. R. [illegible]08). Thimerosal exposure in infants and neurodevelopmental disorders: An as[illegible]ent [illegible]f computerized medical records in the vaccine safety datalink. *Journal of the Ne[illegible]ologi[illegible] Sciences, 271*(1–2), 110–118.

Zhang, L., & Wong, M. H. (2007). En[illegible]mental [illegible]ercury contamination in China: Sources and impacts. *Environment International, 3[illegible]*(1), 10[illegible]121.

RETRACTED ARTICLE
